# The effects of thermal acclimation on lethal temperatures and critical thermal limits in the green vegetable bug, *Nezara viridula* (L.) (Hemiptera: Pentatomidae)

**DOI:** 10.3389/fphys.2012.00465

**Published:** 2012-12-12

**Authors:** Pol Chanthy, Robert J. Martin, Robin V. Gunning, Nigel R. Andrew

**Affiliations:** ^1^Centre for Behavioural and Physiological Ecology, Department of Zoology, School of Environmental and Rural Sciences, Faculty of Arts and Sciences, University of New EnglandArmidale, NSW, Australia; ^2^Cambodian Agricultural Research and Development InstitutePhnom Penh, Cambodia; ^3^Agricultural Systems Research Cambodia Co. Ltd.Battambang, Cambodia; ^4^Maddox Jolie-Pitt Foundation, Rotanak CommuneBattambang, Cambodia; ^5^NSW Department of Primary Industries, Tamworth Agricultural InstituteCalala, NSW, Australia

**Keywords:** *Nezara viridula*, thermal tolerance, lethal temperature, critical thermal limits, acclimation temperature

## Abstract

According to geographical distribution, *Nezara viridula* (Heteroptera: Pentatomidae) can be found across tropical, subtropical, and temperate regions and this pattern is assumed to reflect differences in thermal adaptation, particularly in cold tolerance. Here the lethal temperature (LT) and critical thermal limits (CTL) (thermal tolerance) are examined for *N. viridula*. The upper LT for *N. viridula* at two contrasting climate locations (Breeza and Grafton, New South Wales, Australia) was 40.3°C with 20% survival under the stress of high temperature. The lower LT did not differ between these two populations and was −8.0°C with 20% survival under low temperature stress. Survival of *N. viridula* increased after acclimation at high temperature for 7 days. In contrast, when acclimated at lower temperatures (10 and 15°C), survival of Breeza and Grafton *N. viridula* was lower than 20% at −8.0°C. Control-reared *N. viridula* adults (25°C) had a mean CT_MinOnset_ (cold stupor) of 1.3 ± 2.1°C and a mean CT_Max_ (heat coma) of 45.9 ± 0.9°C. After 7 days of acclimation at 10, 20, 30, or 35°C, *N. viridula* adults exhibited a 1°C change in CT_Max_ and a ~1.5°C change in CT_MinOnset_. CT_Max_ and CT_MinOnset_ of Breeza and Grafton *N. viridula* populations did not differ across acclimation temperatures. These results suggest that short-term temperature acclimation is more important than provenance for determining LTs and CTL in *N. viridula*.

## Introduction

The relationships between potentially lethal environmental temperatures and survival of animals have long fascinated physiologists. Ecologically relevant measures of tolerance to potentially lethal temperatures (LT) have been recently reviewed by Terblanche et al. (2011). Ways in which these relationships are modified through time and space has also attracted study (Chen et al., 1990; Watson and Hoffmann, 1996; Terblanche et al., 2005a,b). Despite much focus on species responses to environmental variation through space and time, many taxa and geographic areas remain poorly studied (Terblanche et al., 2005b). Insect responses to high and low temperature are usually decoupled: lower LT limits typically respond more strongly to acclimation and natural selection than upper lethal limits, and it appears that phenotype can account for much of the response shown by insects (Klok and Chown, 2003; Ayrinhac et al., 2004; Hoffmann et al., 2005). Several studies, however, have shown that decoupling of upper and lower lethal limits is not typical of all insect species and as most investigations are undertaken using model species, this leaves several groups and geographic regions under-represented (Chown et al., 2002; Klok and Chown, 2003). Insects can increase resistance to cold stress when exposed to non-LT conditions prior to the cold stress being applied; these plastic responses are normally described only in terms of immediate effects on mortality (Rako and Hoffmann, 2006). Traditionally, studies of cold tolerance in insects have focused on seasonal adaptations related to overwintering observed after weeks or months of exposure to low temperature. In contrast, an extremely rapid cold-hardening response was observed in non-overwintering stages of insects and conferred protection against injury due to cold shock at temperatures above the supercooling point (SCP) (Lee et al., 1987). In studies of insect cold-hardiness, the SCP is defined as the temperature at which spontaneous nucleation of body fluids occurs (Salt, 1961; Zachariassen, 1985; Czajka and Lee, 1990; Carrillo et al., 2005).

For terrestrial insects, temperature has long been recognized as a major environmental factor responsible for species abundance and geographical distribution. Ambient temperature varies according to daylength and season, so that natural populations are often exposed to heat or cold stress (Gibbs et al., 2003). The capacity to adapt to and tolerate such stresses is extremely important for the persistence of populations (Klok and Chown, 2003; Ayrinhac et al., 2004). In temperate regions, species must tolerate cold conditions during winter and have developed a diversity of adaptive mechanisms to do so, including the occurrence of diapause and the production of antifreeze compounds (Graham et al., 2000; Ayrinhac et al., 2004).

*Nezara viridula* (L.) (Heteroptera: Pentatomidae) is widely distributed across the tropical, subtropical and temperate regions of Eurasia, Africa, Australia, and the Americas (Yukawa and Kiritani, 1965; Yukawa et al., 2007). This distribution pattern is apparently responding rapidly to climate warming (Musolin and Numata, 2003a,b; Musolin et al., 2004, 2007; Kiritani, 2006, 2007; Yukawa et al., 2007), and is especially assumed to be reflected in differences in thermal adaptation, and cold tolerance.

The aim of this paper is to determine for *N. viridula*: (1) whether the LT of populations differs between the two different climatic locations; (2) whether acclimation increases or decreases survival LT; and (3) the effect of acclimation on the critical thermal limits (CTL).

## Materials and methods

### Study site, collection, and maintenance

Samples of the polyphagous green vegetable bug (GVB) (*N. viridula* (L.) were collected from two different areas, Breeza (31°14′54″N 150°28′02″E) and Grafton (29°41′34″N 152°55′56″E), New South Wales, Australia. Breeza represents a dry climate (621 mm annual average rainfall) compared to Grafton (1073 mm). Grafton is also more humid with average annual 3 pm relative humidity of 53% compared to 46% at Breeza. Average annual maximum temperature is similar at both sites (26°C) but minimum temperature is higher at Grafton (13.7°C) compared to Breeza (10.9°C) (BOM, 2011).

Adults were collected on soybean crops by sweep net or beat sheet and placed into plastic containers (64 mm deep and 118 mm in diameter). Air supply was provided by cutting a small hole (65 mm of diameter) in the lid and fitted with mosquito netting. The GVB were provided with fresh green fruit of legume plus water via a cotton wick. All field-fresh GVB samples were returned to the laboratory culture room and maintained at a temperature of 25 ± 1°C, under photoperiodic conditions of light:dark (L:D) 14:10 h and at 60 ± 10% relative humidity. Adult GVB were kept in a cage with 100–150 GVB per cage and fed with fresh green beans (*Phaseolus vulgaris*) plus water supplied via a cotton wick. For acclimation groups, cages were randomly assigned to incubators with temperature regulated at either 10, 20, 30, or 35°C for 7 days with photoperiodic conditions, L:D 14:10 h, before being tested for the effects of acclimated treatments on LTs and CTL. After completion of the acclimation period, the 10–14 day old GVB adults (proportion of male and females approximately 1:1) were taken from the cages and tested immediately. For logistical reasons, the acclimation treatments of two populations, from Breeza and Grafton were tested at the same time. Rearing, acclimation, and experiments were conducted in the Insect Ecology Laboratory of the Zoology Department at the University of New England.

### Lethal temperature of GVB and effects of acclimation temperature on survival lethal temperature

GVB from both Grafton and Breeza were tested to determine whether the LT differed between populations. The lethal (discriminating) temperature was defined as the temperature at which approximately 20% of insects survived for 24 h after a direct plunge to the stress temperature for 2 h (Powell and Bale, 2004; Terblanche et al., 2005b). A group of five GVB adults was placed into a 35 ml vial with enclosing cap. Each treatment temperature was replicated four times. The vials were then placed into double plastic bags with a vial in contact with the thermocouple temperature (Squirrel Data Logger, Grant 2020 series). Thereafter, the plastic bags were submerged in liquid (1:1 water:glycol) in a water bath (Grant R4, Grant instruments, Cambridge, UK) for 2 h set to six temperatures between 36 and 46°C for high temperature stress and set to six temperatures between 0 and −12°C for low temperature stress. The temperature of the thermocouple was recorded every 30 min. It was assumed that the body temperatures of GVB were equivalent to the inside vial temperature. After 2 h treatment, the vials were removed from the water bath and retained in the culture room for 24 h for survival assessment. The GVB were classified as alive if they displayed a righting response and were capable of coordinated walking; and as dead if they were unable to return to their normal upright position or maintained muscle function was lost during the decline in temperature.

The effects of acclimation on survival LT were determined for acclimated individuals in different temperature regimes. Adult GVB were maintained in incubators at 10, 20, 30, and 35°C for 7 days before being tested. Similar methods to those described above were used. A group of five individuals was placed into a vial with a volume of 35 ml and enclosing cap. Each treatment temperature was replicated four times. The vials were then placed into double plastic bags with a vial connected to thermocouple temperature recorders. Thereafter, the plastic bags were submerged in a water bath for 2 h. Thermocouple temperature was recorded every 30 min. After 2 h of treatment, all the vials were removed from plastic bags and placed in the culture room for 24 h to assess survival and mortality of the GVB.

### Critical thermal limits (CTL) and the effects of acclimation temperature on CTL

Upper and lower thermal limits can be measured in different ways such as mortality assays and estimates of knockdown temperature (Hoffmann et al., 1997; Berrigan and Hoffmann, 1998). Knockdown temperature is the upper or lower temperature at which the individuals lose the ability to cling to an inclined surface (Gilchrist and Huey, 1999). Knockdown temperatures were assessed because they are thought to be more ecologically relevant to mobile stages, such as insect adults compared to mortality assays (Hoffmann et al., 1997; Sørensen and Loeschcke, 2001). To determine lower and upper knockdown temperatures, CTL were used. For the low temperature tolerance, critical thermal minima (CT_Min_) were used. These were identified as the onset of chill coma. For the high temperature tolerance, critical thermal maxima (CT_Max_) were used and identified as the onset of heat stupor (Klok and Chown, 1997, 1998).

For this experiment, the methods used were similar to those described by Klok and Chown (2003) and Terblanche et al. (2005b). A group of 10 GVB was assessed for each treatment, and wherever possible three CT_Min_ and CT_Max_ trials were conducted as replications for each acclimation treatment and using the culture-room GVB as control treatment. Ten GVB were placed individually into plastic vials. A copper-constantan thermocouple gauge was placed inside one of the vials to measure vial ambient temperature. It was assumed that the body temperatures of GVB were similar to the temperature inside the vial. The vials were submerged in a water bath which contained distilled water and glycol in a proportion of 1:1. For CT_MinOnset_, the water bath temperature was allowed to stabilize for 15 min at 10°C after which it was lowered at 0.25°C min^−1^ until the onset of chill coma was observed in all individuals. The onset of chill coma was considered as the temperature at which a GVB was unable to right itself. Thereafter, the bath temperature was lowered 0.5°C below the last CT_MinOnset_ value measured and held for 5 min at this temperature before being raised again at 0.25°C min^−1^, the water bath was programmed to increase temperature at 0.25°C min^−1^. As the temperature increased, the behavior of GVB was monitored again for the recovery from cold stupor. The critical thermal minimum recovery (CT_MinRecovery_) was observed until all GVB had regained full motor coordination. The critical thermal maximum (CT_Max_) of individuals was recorded as the onset of muscle spasms during increasing temperature (Lutterschmidt and Hutchison, 1997; Terblanche et al., 2005b). This procedure was continued until the CT_Max_ was reached for all individuals.

### Data analysis

The data sets of the LTs were assessed for probability of survival by using Predictive Analytics SoftWare (PASW) Statistics 18 (SPSS Inc) and curves of LT were generated using the SigmaPlot for Windows 8.02 program (SPSS Inc). The data set of knock down temperatures was examined using a 2-way interaction analysis of variance with IRRISTAT for Windows 5.0 (International Rice Research Institute, IRRI). Data means were compared using least significant differences at the level of 5% (5% LSD).

## Results

### Lethal temperature of GVB and effects of acclimation temperature on survival lethal temperature

**Figure 1:**
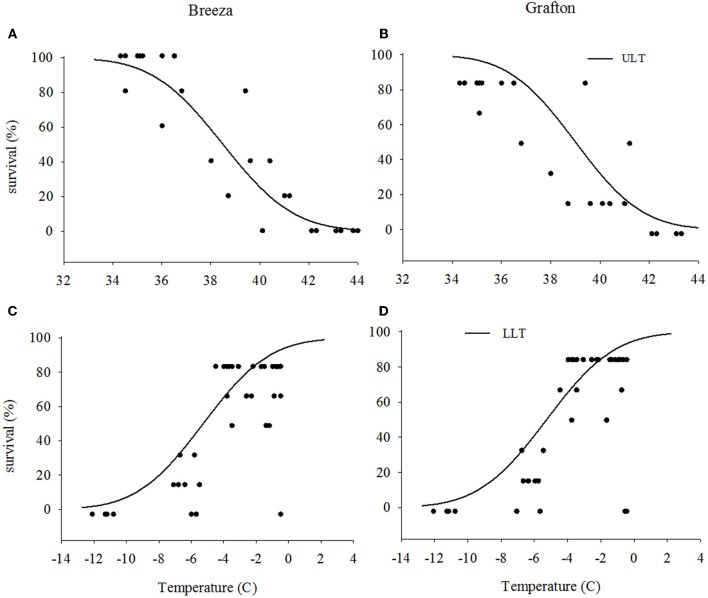
**Upper and lower lethal temperatures (ULT and LLT) of *Nezara viridula* (L.) populations from Breeza (A and C) and Grafton (B and D) for the high and low temperature (the distribution of dots are represented raw data)**.

The upper LT of Grafton GVB (40.4°C) for the high temperature exposure was similar to those from Breeza (40.2°C) (Figures 1A and B) with a mean upper LT of 40.3°C. For the low temperature exposure, lower LT for Grafton and Breeza GVB did not differ (−8.0°C) (Figures 1C and D).

**Figure 2:**
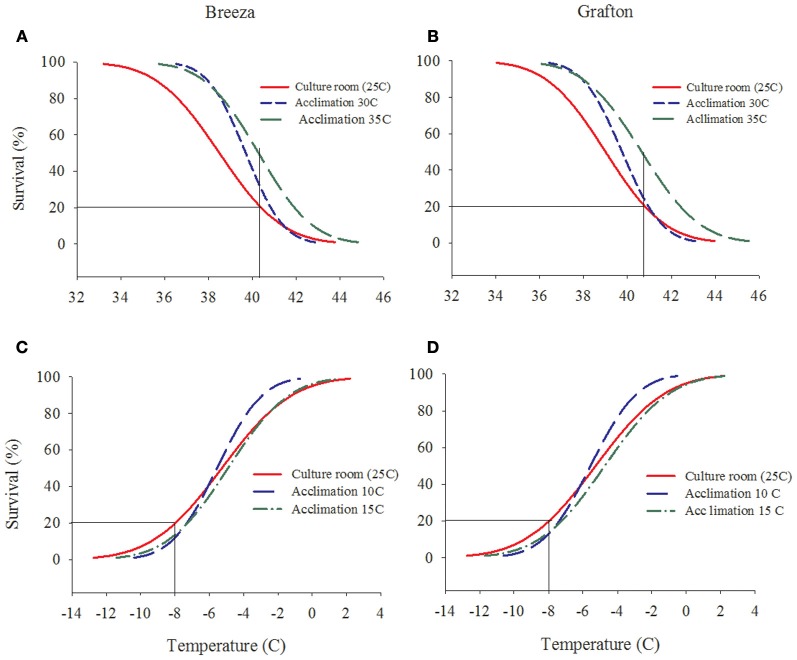
**The effects of acclimation temperature on the upper and lower lethal temperature of *Nezara viridula* (L.) population from Breeza (A and C) and Grafton (B and D) (raw data omitted for clarity)**.

The survival rate of both Breeza and Grafton GVB populations increased by 10–30% when acclimated to higher temperatures of 30°C and 35°C for 7 days compared to GVB maintained at the culture room temperature of 25°C (20% survival at 40.3°C) (Figures 2A and B). However, when Breeza and Grafton GVB populations were acclimated at the low temperatures of 10 and 15°C for 7 days, survival did not improve compared to the 25°C control (20% survival at −8°C). The survival of Breeza and Grafton GVB acclimated at 10°C and 15°C decreased about 10% compared to lower LT for control GVB (Figures 2C and D).

### Critical thermal limits (CTL) and the effects of acclimation temperature on CTL

The 25°C reared GVB control adults had a mean CT_MinOnset_ (cold stupor) of 1.3°C and a mean CT_Max_ (heat coma) of 45.9°C. CT_Max_ did not differ between the 30°C-acclimated, 35°C-acclimated and 25°C control groups (Table 1). However, CT_Max_ of 20°C-acclimated and 10°C-acclimated GVB were significantly different by 0.9°C and 1.1°C compared to the control (*P* < 0.0001). The corresponding differences for the 30°C-acclimated group were 1.2°C and 1.4°C and 1.1°C and 1.3°C for the 35°C-acclimated group (Table 1).

**Table 1:** **Physiological variation of *Nezara viridula* (L.) in culture room and laboratory temperature acclimation**.

**Physiological variable (°C)**	**Acclimation temperature (°C)**					***P***
	**10 (*n* = 60)**	**20 (*n* = 60)**	25^*^(*n* = 60)	**30 (*n* = 60)**	**35 (*n* = 60)**	
CT_Max_	44.8 ± 2.0^b^	45.0 ± 1.0^b^	45.9 ± 0.9^a^	46.2 ± 1.0^a^	46.1 ± 1.6^a^	<0.0001
CT_MinOnset_	0.2 ± 2.0^d^	0.7 ± 2.1^cd^	1.3 ± 2.1^c^	2.4 ± 2.7^b^	4.5 ± 3.0^a^	<0.0001
CT_MinRecovery_	17.1 ± 2.0^cd^	17.9 ± 2.4^c^	16.4 ± 1.7^d^	18.9 ± 2.9^b^	21.5 ± 3.4^a^	<0.0001

*n – number of insects tested*,

“*” insects from culture, 25°C.

Mean ± standard deviation (SD), Means followed by same superscript letters in the same row are not significantly different at p = 0.05 using IRRISTAT program for Windows 5.0. Means were compared by the method of least significant differences at 5% level (5%LSD).

CT_MinOnset_ of the 25°C reared (control) and 20°C-acclimated GVB did not differ significantly, but were significantly lower than the CT_MinOnset_ for GVB acclimated to 30 and 35°C. CT_MinOnset_ of 10°C-acclimated GVB was significantly lower (1.1°C) than that of the controls, 30°C-acclimated GVB (2.2°C), and 35°C-acclimated GVB (4.3°C). CT_MinOnset_ of 10°C-acclimated and 20°C-acclimate GVB did not differ significantly (Table 1). CT_MinRecovery_ tended to be higher in GVB exposed to the higher temperatures, but the patterns were unclear as they were exposed to lower treatment temperature compared to the 25°C control (Table 1). CT_MinRecovery_ of 30°C-acclimated and 35°C-acclimated GVB were significantly higher by 2.5°C and 5.1°C respectively compared to those of 25°C controls (Table 1). Overall, CT_Max_ varied by about 1°C over the treatment temperatures, whereas variation was slightly higher for CT_MinOnset_.

CT_MinRecovery_ of Breeza 30°C-acclimated and 35°C-acclimated GVB was significantly higher than the corresponding data from Grafton GVB (1.4 and 2.1°C, respectively). CT_MinRecovery_ of other acclimated temperatures did not differ between Breeza and Grafton (Table 2) and CT_Max_ and CT_MinOnset_ of Breeza and Grafton GVB did not differ across acclimated temperatures (Table 2).

**Table 2:** **Interactions of location and physiological variation of *Nezara viridula* (L.) in culture room and laboratory temperature acclimated**.

**Locations**	**Number of insects tested**	**Acclimation temperature (°C)**	**Physiological variation (°C)**		
			**CT_Max_**	**CT_MinOnset_**	**CT_MinRecovery_**
Breeza	30	10	44.4 ± 2.0 a	0.2 ± 2.2 a	17.1 ± 2.1 cd
Breeza	30	20	45.0 ± 1.2 a	1.0 ± 2.1 a	17.9 ± 2.4 c
Breeza	30	25^*^	45.9 ± 0.8 a	1.2 ± 1.9 a	16.4 ± 2.0 d
Breeza	30	30	45.9 ± 1.1 a	2.2 ± 2.8 a	**19.6 ± 3.7 b**
Breeza	30	35	46.1 ± 1.8 a	4.6 ± 3.1 a	**22.6 ± 4.0 a**
Grafton	30	10	45.2 ± 1.8 a	0.2 ± 1.9 a	17.1 ± 2.0 cd
Grafton	30	20	45.0 ± 0.7 a	0.4 ± 2.1 a	17.9 ± 2.5 c
Grafton	30	25^*^	45.9 ± 1.1 a	1.3 ± 2.2 a	16.5 ± 1.5 d
Grafton	30	30	46.4 ± 0.8 a	2.5 ± 2.6 a	**18.1 ± 1.6 c**
Grafton	30	35	46.1 ± 1.5 a	4.4 ± 3.0 a	**20.5 ± 2.2 b**

Bolded results are significant differences between locations within the same acclimation temperature.

* Insects from culture, 25°C.

Mean ± standard deviation (SD), Means followed by same letters in the same column are not significantly different at p = 0.05 using IRRISTAT program for Windows 5.0. Means were compared by the method of least significant differences at 5% level (5%LSD).

## Discussion

LT (set at 20% survival) is a method that can be used to describe the survival of GVB under unpredictable stress or extreme temperatures that are common within the insect's natural thermal habitat. LT has been shown to be an important measure of survival for a variety of other insect species and provides an indication of ability to survive unexpected and potentially lethal short term declines in temperature (Lee et al., 1987; Kelty and Lee, 1999; Terblanche et al., 2005b). LT of Breeza and Grafton GVB for the high temperature range averaged 40.3°C (40.2°C for Breeza and 40.4°C for Grafton) and for the low temperatures it was −8.0°C for both populations. These results might be an indication that the climatic differences between Breeza and Grafton were insufficient to affect LTs of GVB. Breeza represents a dry inland climate (621 mm annual average rainfall) compared to coastal Grafton (1073). Grafton is also more humid with an average annual 3 pm relative humidity of 53% compared to 46% at Breeza (Gunnedah). Though, the average annual maximum temperature is similar at both sites (26°C), the average minimum temperature is higher at Grafton (13.7°C) compared to Breeza (10.9°C) (BOM, 2011). The small variation of temperature between these two habitats did not result in significant LT effects between GVB populations. Moreover, after collection of GVB from both locations, the insects were maintained in the same culture room under the same climatic conditions: temperature of 25°C and relative humidity of 60% for 7 days. Acclimation for 7 days at 10 and 15°C decreased survival of GVB by ~10% (range 10–20%) compared to non-acclimatised survival of LT (Figures 2C and D). Previous studies on other insects suggest that acclimation for longer times than the period required to induce maximum cold hardening resulted in slight decreases in survival with every extra hour of acclimation, indicating that extended exposure to low temperatures increases mortality (Powell and Bale, 2004). However, acclimation at 0°C for two and 3 h for nymphs and adults of the grain aphid, *Sitobion avenae* (Homoptera: Aphididae), respectively increased nymphal survival from 18 to 83% and adult survival from 16 to 68% compared with nymphs and adults exposed directly to the LT (Powell and Bale, 2004, 2005). In the present experiments, however, when GVB were acclimated at nonlethal higher temperatures of 30 and 35°C, LT survival increased by up to 50% (Figures 2A and B). These results suggested that Breeza and Grafton GVB were tolerant to warmer than average temperatures and they would adapt to the temperature extremes in the environment for survival.

Chen et al. (1990) revealed that adaptations to temperature stress differ between tropical and temperate flesh flies (Diptera: Sarcophagidae). While sarcophagids from both geographical areas share a mechanism for rapidly increasing heat tolerance only temperate zone sarcophagids appeared capable of responding rapidly to cold stress. One consistent parameter to change with thermal acclimation in ectotherms is the ability to withstand extreme temperature, warmer acclimated animal will have a higher tolerance to high temperature exposure than the same animal when cold acclimated (Cossins and Bowler, 1987). It is important to note that the daily maximum and minimum temperature in 2010 at Breeza and Grafton did not exceed the upper and lower LT of GVB. At Breeza in 2011 there were 2 days (26–27 January 2011), which the daily maximum temperature exceeded the upper LT of GVB. However, the daily maximum temperature in Grafton did not exceed the ULT of GVB. The minimum temperature in 2010 and 2011 did not exceed the lower LT neither locations (BOM, 2012). In *Drosophila* resistance to high temperature has been much studied as a trait subject to selection (Levins, 1969; Quintana and Prevosti, 1990; Huey et al., 1992; Krebs and Loeschcke, 1997).

Insects can increase resistance to heat or cold stress when exposed to non-lethal conditions prior to stress; these plastic responses are normally described only in terms of immediate effects on mortality (Rako and Hoffmann, 2006). From the present GVB data, acclimation at temperatures lower than the 25°C control had a strong, significant and negative impact on the CT_Max_. Overall, the mean temperature for thermal tolerance (CT_Max_ – CT_MinOnset_) was 43.8°C (ranging from 41.6 to 44.6°C depending on the acclimation temperature). At Breeza and Grafton, the daily maximum temperature in 2010 was below 40°C as mentioned earlier. However, based on long-term climate records, the highest daily temperatures in the Breeza area have ranged from 43.3 to 48.7°C and at up to 43.8°C at Grafton (BOM, 2012). These extreme temperatures exceeded the thermal tolerance of GVB and may have affected the mortality rate of GVB at both locations. In NSW, summer temperature is expected to rise by up to 2.3°C by 2030 as a result of climate change (CSIRO, 2006). It is therefore expected that GVB will be exposed to lethal high temperature extremes more frequently in the future, particularly at Breeza.

Temperate insects have a consistent pattern of thermal responses (Chen et al., 1990; Chown, 2001; Klok and Chown, 2003). In GVB, CT_Max_ declined significantly with low temperature acclimation and increased with high temperature acclimation, but did not differ significantly compared to the constant 25°C control (Table 1). The difference in CT_Max_ between all the treatments was more than 1°C, whereas the CT_MinOnset_ temperatures were significantly affected by acclimation, spanning a range of 1.5°C over an acclimation range of 10–35°C. These results are broadly consistent with other insect observations that, upper and lower CTL are typically decoupled, with lower critical limits showing great variation and a more plastic response to acclimation than upper limits (Chown, 2001; Kimura, 2004; Hoffmann et al., 2005; Terblanche et al., 2005b).

**Figure 3:**
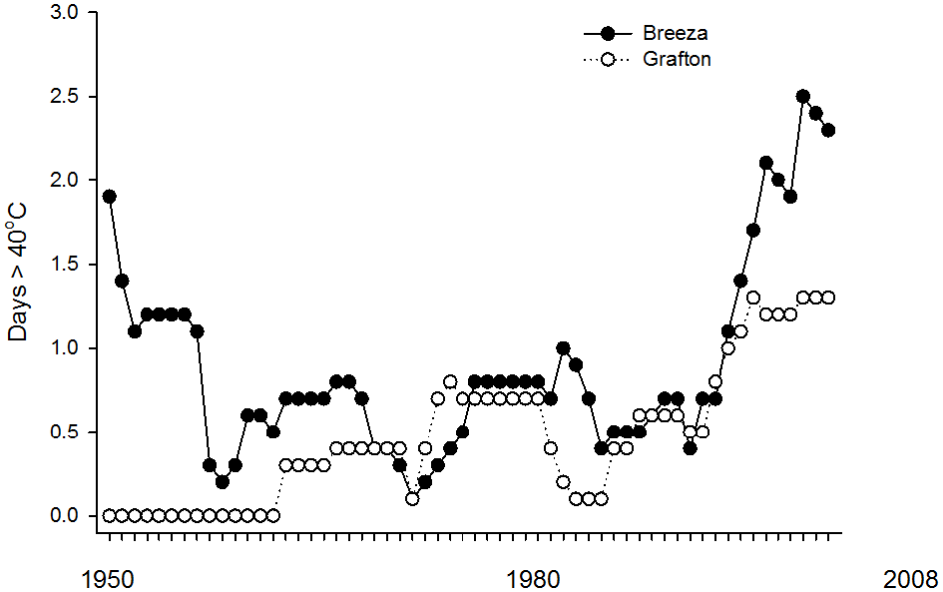
**Ten-year moving averages for number of days per year exceeding 40°C based on historical temperature (1950–2008) at Breeza and Grafton**.

CT_Max_ and CT_MinOnset_ did not differ between populations of Breeza and Grafton GVB. Grafton is also more humid with average annual 3pm relative humidity of 53% compared to 46% at Breeza. Average annual maximum temperature is similar at both sites (26°C) but minimum temperature is higher at Grafton (13.7°C) compared to Breeza (10.9°C) (BOM, 2011). There is evidence from Global observations gathered since 1950 of change in some climate extremes. Global-scale trends in extremes for temperature are considered to be reliable but regional-scale trends could be less reliable depending on the geographical uniformity (IPCC, 2012). Based on historical temperature records from 1950 (BOM, 2012), there have been 62 days exceeding 40°C at Breeza (1.0 days per year). However since 1990, there have been 35 days exceeding 40°C at Breeza (1.9 days per year) (Figure 3).

In comparison, there are fewer high temperature extremes at Grafton. Since 1950, there have been 30 days exceeding 40°C at Grafton (0.5 days per year). Since 1990, there have been 19 days exceeding 40°C at Grafton (1.0 days per year).

The occurrence of days exceeding 40°C at both Breeza and Grafton has been increasing since 1990 (Figure 3). However, records at Breeza show that it has not followed the global trend for increasing temperatures since the 1890s where, at Breeza, 65% of the days exceeding 40°C were recorded between 1899 and 1950 with 6.6 days per year exceeding 40°C during this period. Therefore regional-scale climate trends appear to be operating at Breeza.

Overall, Breeza appears to be a less hospitable place for *N. viridula* than Grafton with respect to temperatures exceeding 40°C. If the current trend for increasing frequency of temperatures exceeding 40°C at Breeza continues, conditions could become unsuitable for *N. viridula*, especially if they return to the levels experienced between 1899 and 1950. The number of days per year below −8°C has not occurred at either location.

In summary, data presented here suggest that the LT did not differ between two populations of GVB collected at Breeza and Grafton. LTs for GVB were shown to be 40.3°C and −8.0°C for high and low temperatures, respectively. *N. viridula* responded more strongly to extreme hot temperature, survival was increased at the upper LT when GVB were acclimated at 30 or 35°C for 7 days. In contrast, survival of GVB decreased for lower LT when acclimated at 20 or 10°C. Therefore, when knock-down of GVB under extreme temperature was investigated, CT_Max_ of GVB did not differ between those maintained at constant 25°C control and high temperature acclimated (30 or 35°C) groups. CT_MinOnset_ of 10°C-acclimated GVB was significantly lower (by 1.1°C) than that of GVB from the 25°C constant temperature control. These data suggest that GVB species are more tolerant of low temperature when acclimated at 10°C.

Much remains to be learned about the mechanistic basis and ecological and physiological relevance of LT for *N. viridula*. Further study is required to determine LT of nymphal stages, GVB response to recovery time after exposure to extreme temperature and rapid cold hardening (RCH). RCH in insects is detected as an increase in survival at a LT following a brief period of acclimation (1–3 h), at low temperature (typically 0°C) (Powell and Bale, 2005). Finally, a better understanding of desiccation resistance in nymphs and adult GVB would be helpful in predicting response to climate change.

### Conflict of interest statement

The authors declare that the research was conducted in the absence of any commercial or financial relationships that could be construed as a potential conflict of interest.
